# The Overlooked Association Between Nutrition and the Development of Acute Myeloid Leukaemia: A Scoping Review

**DOI:** 10.1007/s13668-024-00522-2

**Published:** 2024-03-02

**Authors:** Alex Rezae, Reem Fakak, Kyle G. Alexander, Constantina Constantinou

**Affiliations:** Department of Basic and Clinical Sciences, University of Nicosia Medical School, 21 Ilia Papakyriakou, 2414 Engomi P.O. Box 24005, CY-1700 Nicosia, Cyprus

**Keywords:** Acute Myeloid Leukaemia, Nutrition, Risk factors, Prevention, Red meat

## Abstract

**Purpose of Review:**

Acute Myeloid Leukaemia (AML) constitutes a mere 1% of global cancer cases. This scoping review aims to investigate the association between nutrition and the development of AML, providing a foundation for future research in this field.

**Recent Findings:**

A systematic search was conducted across PubMed, EBSCO, Taylor and Francis, Science Direct and Cochrane Library using specific keywords. Inclusion criteria comprised observational studies and clinical trials examining the association between nutrition and the development of AML. Articles selected for analysis were restricted to those published in English between 1990-2023, and available as full text articles. Among the twenty-five articles that were screened, only six met the criteria for data extraction. Four studies did not reveal statistically significant correlations between nutrition and the development of AML, while two studies provided evidence for significant associations. The findings indicated increased AML risk associated with (a) heightened caloric intake, consumption of white potatoes, and red meat (pork and beef) and (b) diminished consumption of vegetables, seafood, nuts, and seeds.

**Summary:**

The scarcity of comprehensive studies exploring the connection between nutrition and AML, highlights the urgent need for additional research, encompassing pre-clinical studies. This imperative is critical for enhancing our understanding of the molecular mechanisms that underlie the connection between diet and the aetiology of AML. Such knowledge is paramount in advancing effective strategies for both prevention and management of this significant disease.

## Introduction

Acute myeloid leukaemia (AML) originates from myeloid precursor cells and constitutes approximately 1% of all cancer cases and 23% of all leukaemia cases worldwide [1, 2]. AML typically affects older individuals, primarily females, with a median age of onset of 68 years; yet it also has a notable prevalence in the paediatric population [3, 4]. In 2023, the estimated 5-year survival rate of AML patients in the United States was 28% for individuals over 20 years-old and 69% for those below the age of 20 [3].

Chromosomal translocations and mutations are implicated in the causation of AML [5]. AML is classified based on the French-American-British (FAB) classification ‘M0 -M7’ which describes the morphology and maturation of myeloblasts. Subtypes M0-M5 are characterised by mutations in immature leukocytes, while M6 and M7 contribute to acute erythroid leukaemia and acute megakaryoblastic leukaemia, respectively [6]. Genetic mutations in haematopoietic stem cells, coupled with lifestyle factors such as smoking, obesity, and benzene exposure, contribute to the carcinogenic processes leading to the development of AML [7].

The World Cancer Research Fund (WCRF) published the third edition of the Diet and Cancer Report in 2018, aiming to guide individuals in making informed decisions to mitigate cancer risk through healthier dietary choices, weight management, and enhanced physical activity [8•]. The guidelines underscore the evidence, that consumption of red and processed meats increase the risk for some cancers particularly due to the presence of the highly carcinogenic N-nitroso metabolite [9]. Diets associated with a reduced risk of cancer contain less than modest amounts of red meat and little to no processed meat (≤ 350–500 g). WRCF advocates for a diet rich in wholegrains, vegetables, fruits, legumes and a daily fibre intake of 30 g. Notably, the Diet and Cancer Report highlights evidence associating consumption of vegetables with a decreased risk of leukaemia; yet research between the association of leukaemia and nutrition is relatively limited compared to studies on other cancer types [8•].

In vitro studies have provided evidence for the anti-proliferative capacity of specific nutrients in AML cell lines. For instance, curcumin, the polyphenol found in turmeric, inhibits growth of AML cells and induces apoptosis through targeting of the AKT/PKB pathway [10]. This also proposes the potential use of curcumin adjunct to afuresertib (an AKT inhibitor), which can synergistically suppress AML proliferation. Several other polyphenols, including resveratrol found in wine, epigallocatechin-3-gallate found in green tea, genistein found in soybeans, lycopene and quercetin, have all demonstrated anti-leukemic effects against AML cells [11]. Hence, specific nutrients derived from certain foods exhibit established anti-proliferative effects on AML cells. Further research in this domain holds the potential to elucidate the role of dietary factors as risk factor for AML.

Vitamin D plays a crucial role in regulating cell growth, differentiation, and apoptosis, and is primarily obtained through sunlight exposure and foods such as fatty fish and fortified dairy products. Deficiency of vitamin D has been associated with the development of AML. Specifically, one study has shown that a high prevalence of vitamin D deficiency was found among patients newly diagnosed with AML, and AML patients deficient in vitamin D had a reduced overall survival when compared to patients with sufficient vitamin D levels [12•].

Fatty fish, flaxseeds, and walnuts are a good source of lipid mediators such as arachidonic acid and omega-3 fatty acids. Lipid mediators influence inflammation, immune responses, and cell signalling and have been discussed as beneficial supplements during AML treatment [13]. However, the effect of omega-3 supplementations on AML incidence has yet to be established.

Studies in the literature have investigated an association between nutrition and childhood leukaemia. Some studies suggest that obesity and malnutrition are associated with poorer survival rates in children with AML [14, 15]. Additionally, the consumption of fresh fruits and vegetables during pregnancy have been associated with a decreased risk of infant leukaemia, specifically AML [16, 17]. This remains a relatively unexplored avenue, underscoring the importance of further research into the association between nutrition and AML risk.

The objective of this scoping review is to investigate the association between nutrition and the risk of developing AML in adults.

## Methodology

This scoping review was conducted based on the methodological framework developed by key authors in the field andll the steps comply with the most recent relevant guidance [18, 19].

### Research Question

Our research question is:

- Is there an association between nutrition, dietary intake and the risk of developing AML?

### Search Strategy

For this scoping review we conducted a search using the electronic databases PubMed, EBSCO, Science Direct/Elsevier, Taylor and Francis and Cochrane Library, for articles that were written in the English language and published during the period of 1990-2023 and were available as full text articles. An extensive time frame was searched with the aim of providing insight into the historical context of AML and nutrition and to identify gaps in the research.

The aim of the review was to identify primary research studies that investigated the association between nutrition and the risk of developing AML. In the above databases, we used the following keywords and applied Boolean operators using the MeSH generator tool. We conducted the following search: (“Acute myeloid leukaemia”) AND (“Nutrition” OR “Diet” OR “Dietary habits” OR “total caloric intake” OR “macronutrients” OR “micronutrients” OR “Vegetables” OR “Grains” OR “Cereals” OR “Wholegrains” OR “fruits”, OR “legumes”, OR “dairy products” OR “Seafood” OR “Fish” OR “Poultry” OR “Chicken” OR “Red meat” OR “Beef” OR “olive oil” OR “Salt”).

### Inclusion Criteria

The primary inclusion criteria were studies examining the association between diet and AML, published during the period of 1993-2023 in the English language and for whom full text articles were available. More specifically, our aim was to include both primary (cross-sectional, case control, retrospective and prospective studies and randomised control trials) and secondary studies (systematic reviews/meta-analysis) that explored the association between nutrition and risk for developing AML in adults between ages 18-90. Additional information for the current paper was extracted from NIH (National Library of Medicine), American Association for Cancer Research, Springer Link and Oxford Academic.

### Exclusion Criteria

We excluded articles not published in the English language and published prior to 1993. Additionally, we excluded studies not aligned with our research aim, in vitro studies, inaccessible studies, studies on pregnant women, and studies focusing on the progression rather than the incidence of leukaemia. Additionally, we excluded studies that were not original journal articles, such as conference notes, narrative reviews, or studies that were not accessible in full text.

### Data Extraction

Data extraction and validation were carried out independently by two researchers (RF and AR). The following data were extracted: author name, year, study type, country in which the study was conducted, study aim, age range of the participants in the study, study type, key findings, and conclusions (Table 1).

**Table 1:** Studies reporting no statistically significant association between nutrition and AML

Study	Year	Aim	Type of study	Country	Participants	Methods	Key findings	Conclusions
**Cross sectional study**								
**Hursting et al. **[20••]	1993	To explore the relationship between dietary factors and acute myeloid leukaemia	Cross-sectional study	Australia, Canada, Colombia, Denmark, Finland, France, Hong Kong, Iceland, Israel, Italy, Japan, Netherlands, New Zealand, Norway, Philippines, Romania, Spain, Sweden, Switzerland, United Kingdom, United States, West Germany, Yugoslavia	Women and men ages 35–64 from all five continents	• Statistical analysis of international food supply data correlated with all leukaemia subtype incidences • The leukaemia incidence was collected from 24 countries cancer registries • Leukaemia registries were regressed with per capita macronutrient deficiency as well as Gross national product, height, and alcohol	• The association between total calorie intake and risk of AML was weak in male (P* = 0.19) and moderate in females (P = 0.36) • Total dietary fat intake and risk of AML in males was weak (P = 0.43) and moderate in females (P = 0.56) • Dietary carbohydrate intake and risk of AML showed a moderate inverse relationship in female (P = -0.48) and male (P = -0.49) • Total dietary protein consumption in male (P = 0.17) and female (P = 0.27) • was weakly associated to AML risk and showed no statistically significant risk • *Pearson correlation coefficient	• The association between total calorie intake and risk of AML was weak in males and moderate in females but was not statistically significant • Total dietary fat intake was associated with a mild to moderate correlation to AML risk in both genders but not statistically significant • Carbohydrate intake was associated with a lower risk of AML in both males and females but was not statistically significant • Total dietary protein consumption in males and females was weakly associated to increased AML risk but was not statistically significant
**Cohort studies**								
**Ross et al.** [21••]	2002	To examine the association between consumption of vegetables and the development of AML	Cohort study	Iowa, USA	41,836 women (ages 55–69)	• Baseline questionnaires were administered to participants to assess total meat intake: beef, poultry, fish, pork, and processed meats • Follow-up: 8 years. Vital status ascertained through social security. Cancer diagnosis established through cancer registry	• Of the cohort of women between ages 55–69, 138 developed AML from the period 1986–1999 in the state of Iowa • Increased vegetable consumption was associated with decreased risk of AML. (P = 0.10) • Increased risk AML for those women had increased consumption of milk (P = 0.79 for skim milk and P = 0.64 for whole milk) • Increased risk of AML for women that increased consumption of soft water • Increased risk AML for those women that had increased consumption of poultry (P = 0.89 for chicken with skin, P = 0.43 for chicken without skin)	• Women who developed AML had lower vegetable consumption, compared to healthy women. This was not statistically significant • Increased consumption of milk, soft water and poultry led to a non-statistically significant increase in AML association
**Ma et al. **[22••]	2009	To assess the possible aetiologic role of dietary factors in AML in the NIH-AARP Diet and Health study	Cohort study	8 US states (California, Florida, Louisiana, New Jersey, North Carolina, Pennsylvania, Georgia, and Michigan)	491,163 women and men ages 50–71	• Baseline questionnaires were administered to participants to assess total meat intake: beef, poultry, fish, pork, and processed meats • Follow-up: 8 years • Vital status ascertained through social security. Cancer diagnosis established through Cancer Registry. Patients that were diagnosed with AML in the first one or 2 years were omitted. Follow-up was extended from the years 1995–1996, to date of death, date of AML diagnosis or relocation out of registry delegated area or 2003 December	• Individuals with total meat intake (all types of beef, poultry, fish, pork, and processed meats) higher than 91.8 g per 1000 kcal had increased risk of developing AML • High consumption of total meat was associated with AML risk (HR: 1.45, 95% Cl = 1.02–2.07, • P = 0.06) • No coffee consumption had the highest association with AML. (P = 0.59) • Fruits were not associated with AML. (P = 0.65) • Vegetables were not associated to AML. (P = 0.13)	• Increased association between higher meat intake and the risk of AML, but not statistically significant • Increased association between those who did not drink coffee and AML. This was not statistically significant • Fruits and vegetables did not have any sort of statistically significant association to AML
**Hosnijeh et al. **[23••]	2013	To examine the potential association between dietary factors and leukaemia	Cohort study	Denmark, France, Greece, Germany, Italy, Netherlands, Norway, Spain, Sweden, and United Kingdom	477,325 participants (142,259 men and 335,066 women) ages between 35–70	• Part of larger EPIC study: questionnaires and anthropometric data collection: Gathering blood samples, removing serum, red cells and buffy coat fractions were removed • Participants recalled the diet for the 24 h prior to the blood sample collection • Participants provided information on diet over the past 12 months at recruitment • Follow up of 11.34 years (SD = 2.47) • Outcome assessment in accordance with International Classification of Diseases for Oncology	• Highest (Q2) vs lowest (Q4) quintile of red meat (g/day) (control HR = 1, Q2: HR = 0.99 (95% CI = 0.63–1.55), Q4: HR = 1.1 (95% CI = 0.66–1.84) P = 0.68) (Positive association between red meat and AML risk but not statistically significant) • Highest vs lowest quintile of poultry (g/day (control HR = 1, Q2: HR = 1 (95% CI = 0.64–1.56), Q4: HR = 1.02 (95% CI = 0.65–1.61) P = 0.92) (positive association between poultry and AML risk but not statistically significant) • Highest vs lowest quintile of processed meat (g/day). (Control HR = 1, Q2: HR = 1 (95% CI = 0.71–1.63), Q4: HR = 0.88 (95% CI = 0.53–1.46) P = 0.9 (positive association between processed meat and AML risk but not statistically significant) • Highest vs lowest quintile of Offal (g/day) (Control HR = 1,) Q2: HR = 0.98 (95% CI = 0.71- 1.63), Q4: HR = 0.99 (95% CI = 0.64–1.54), P = 0.62) (positive association between offal and AML risk but not statistically significant result of) • Highest vs lowest quintile of Fish (g/day) (control HR = 1, Q2: HR = 1.52 (95% CI = 0.93–2.48), Q4: HR = 1.75 (95% CI = 1.02–2.97), P = 0.07). (positive association between fish and AML risk but not statistically significant) • Highest vs lowest quintile of Fruit (g/day) (Control HR = 1, Q2: HR = 1.06 (95% CI = 0.7- 1.63), Q4: HR = 1.30. (95% CI = 0.81–2.07), P = 0.36). (positive association between fruit and AML risk but not statistically significant result)) • Highest vs lowest quintile of nuts/seed (g/day) (Control HR = 1), Q2: HR = 0.69 (95% CI = 0.43–1.13), Q4: HR = 1.27 (95% CI = 0.79—2.04), P = 0.76) (No statistically significant association between nuts/seeds and AML risk) • Highest vs lowest quintile of vegetables (g/day) (control HR = 1, Q2: HR = 0.9 (95% CI = 0.63–1.54), Q4: HR = 1.05 (95% CI = 0.63–1.75), P = 0.94). (positive association between vegetables and AML risk but no statistically significant result) • Highest vs lowest quintile of Dairy products (g/day) (Control HR = 1, Q2: HR = 0.99 (95% CI = 0.63—1.54), Q4: HR = 1 (95% CI = 0.64–1.54), P = 0.94 (positive association between dairy products and AML risk but no statistically significant result of)	• The associations between all food (red meat, poultry, processed meat, offal, fish, fruit vegetables and dairy) and AML were not statistically significant

### Data Synthesis

The procedure of data synthesis encompassed the summarising of key findings of the included studies and examination of interrelations among these studies through a narrative approach. In total, 6 studies were included in the review (Table 1).

## Results

**Fig. 1:**
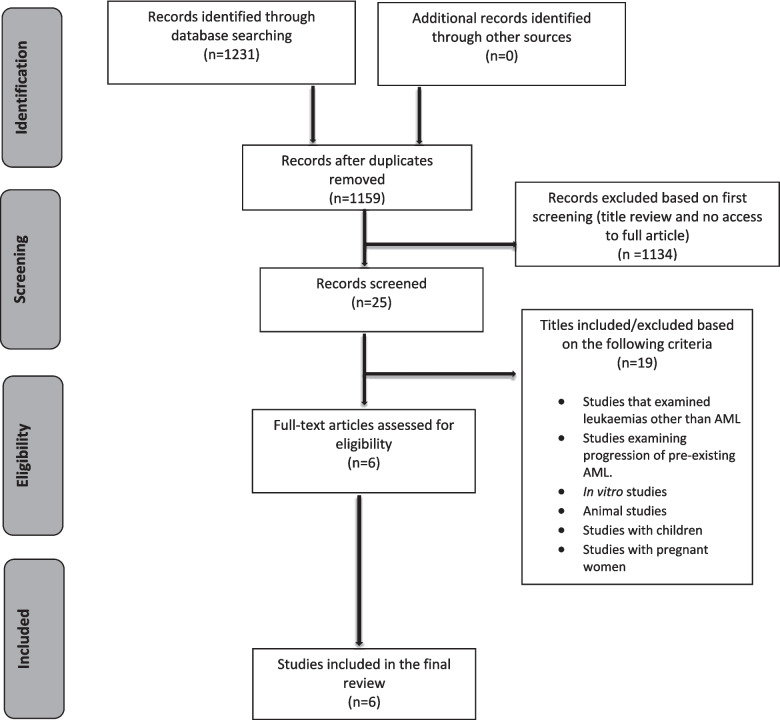
PRISMA flow diagram

The selection process is visualised using a PRISMA flow diagram (Fig. 1). In the original search, a total of 1231 journal articles were retrieved through databases. After removing duplicates and screening titles, the total article count was narrowed down to 25 studies. After reviewing full text articles, 19 of the remaining articles were deemed ineligible as they did not meet one or more of the criteria and therefore six primary articles were analysed and included in the paper. No secondary studies met our inclusion criteria (Fig. 1).

Out of the six studies [20••, 21••, 22••, 23••, 24••, 25••], one was a cross-sectional study [20••], two were case–control studies [24••, 25••], and three were cohort studies [21••, 22••, 23••]. Participants in human studies were recruited from the US in five studies [20••, 21••, 22••, 24••, 25••], from European countries (Denmark, France, Greece, Germany, Italy, Netherlands, Norway, Spain, Sweden, and the United Kingdom) in one study [23••] and 23 countries from five different continents in another study [20••] (Tables 1 and 2).

One study examined the relationship between total caloric intake and macronutrients and the incidence of AML [20••]. Four studies investigated the relationship between specific food groups and AML [21••, 22••, 23••, 24••] (Table 1).

### Studies Reporting No Statistically Significant Association Between Nutrition and AML

Among the five studies investigating the association between nutrition and AML, four studies established a trend that consumption of certain food (red meat, white meat, and fish) or macronutrients increased the risk of AML. However, these results were not statistically significant [20••, 21••, 22••, 23••] (Table 1).

#### Cross Sectional Study

Hursting et al. [20••] conducted a cross sectional study to evaluate the association between dietary factors and the development of AML. The study reported that while the consumption of dietary protein in males and females was weakly associated to AML, it was not statistically significant (Table 1).

#### Cohort Studies

Ross et al. [21••] assessed the relationship between vegetable consumption and risk of AML in the Iowa Women’s Health Study. The study found an inverse association between increased vegetable consumption and risk for leukaemia in women, which however, was not statistically significant (P = 0.08) (Table 1).

Ma et al. [22••] investigated the association between consumption of processed red and white meat and the development of AML. The results of the study indicated that high total meat intake was associated with an increased risk of AML; yet the findings were not statistically significant (P > 0.05) (Table 1).

Hosnijeh et al. [23••] explored the association between large food groups and development of AML in adults (ages 35–70). The study reported a positive association between consumption of red meat, poultry, processed meat, offal, fish, fruit vegetables and dairy and increased risk of AML. Nevertheless, similar to the previous studies, the researchers concluded that the results of this study were not statistically significant (Table 1).

### Studies Reporting a Statistically Significant Association Between Nutrition and AML

#### Case Control Studies

Li et al. [24••] conducted a case -control study in 2006 to assess the association between the risk of AML and various foods, fruits, vegetables and beverages. A protective effect was found between high consumption of tea and decreased risk of AML in both genders. Meanwhile women with diets high in beef, milk, coffee, beer and wine had a significantly increased risk of developing AML. The rest of the examined foods and beverages including vegetables, fruits, processed meat, pork, liquor, total alcohol, decaffeinated coffee, different colas and other soft drinks did not yield statistically significant results [24••] (Table 2).

Yamamura et al. [25••] conducted a case control study in 2013 to examine the association between consumption of vegetables, fruits, and meats with risk of AML. The researchers reported that increased intake of white potatoes, red meat and overall caloric intake was associated with increased risk of AML. Additionally, dark green vegetables, seafoods, nuts and seeds were associated with a lower risk for AML. This study further stratified risk based on sex and concluded that women who consumed more dark-green vegetables, orange vegetables and nuts/legumes had the lowest risk of developing AML. Regarding men, those who consumed the higher quantities of poultry, fruits and seafood experienced the lowest risk of developing AML [25••] (Table 2).

**Table 2:** Studies reporting statistically significant association between diet and AML

Study	Year	Aim	Type of study	Country	Participants	Methods	Key findings	Conclusions
**Li et al. **[24••]	2006	Few studies have explored the association between diet and adult acute myeloid leukaemia (AML)	hospital-based case–control study	Roswell Park Cancer Institute, Buffalo, NY, USA	111 cases (56 men and 55 women) 439 controls (221 men and 218 women)	• Adult Patients in Roswell Park cancer institute between the years 1982–1998 • Patient epidemiology data system Questionnaire was used, food questionnaire was included in this • Controls were patients treated for non-neoplastic conditions • Controls and Cases were matched for age and gender and measured over 5-year strata • Nutrients calculated based on the foods listed on the questionnaire. Regression coefficients were assigned to each food and then multiplied by the use found in the questionnaire	• Women in higher income households had a higher incidence of AML compared to their lower income counterparts. There was no difference for men. (p = 0.037) • Upper and middle tertiles of beef intake had a positive association with AML (P = 0.02) in women only • No positive association for either pork or processed meat for both genders. Nor was it statistically significant • Women that were category with the most alcohol intake, specifically women that consumed beer and wine, had the highest incidence of AML. However only the incidence for wine was statistically significant (P = 0.01). There was no other statistically significant findings for any beverage • Women that were in the middle and upper tertiles of milk consumption had a statistically significant increase of incidence of AML (P = 0.002)	• There are no protective effects of fruits for either gender. • There was a positive association between AML risk and beef intake among women only. • Tea may have a chemoprotective effect for both genders. • There are positive associations for beer and wine intake among females. • Increased coffee intake was observed together with a higher risk of AML. This is despite some of the supposed chemoprotective effects of coffee and is probably caused by topo II inhibitors found in coffee. This was specific to women. • Women that consumed more milk had lower incidence of AML.
**Yamamura** **et al. **[25••]	2013	To examine the association between consumption of vegetables, fruits, and meats with risk of AML	Case control study	Texas, USA	703 men and women between ages of 18–87	• Adult participants from MDACC (MD Andersson Cancer Centre) aged 18–87 that registered between the years 2003–2007 were included; cases were de novo, meaning no history of prior radiation or chemotherapy • Participants completed questionnaires, demographic, risk factors (HHHQ) and food frequency questionnaires	• Obesity reported in (40% of AML cases vs 27% of non-AML cases). P < 0.001 • Caloric intake was higher in AML cases (2536 kcal) than non-AML cases (2293 kcal) P = 0.001 • Both white potatoes and red meat/pork increased risk of AML for both sexes P < 0.01 • Total Vegetables decreased risk of AML (Women: P < 0.01; Men: P = 0.72) • Orange vegetables decreased risk of AML (Women: P < 0.01 Men: P = 0.31) • Dark-green vegetables decreased risk of AML for both sexes (P < 0.01) > • Seafood decreased risk of AML for both sexes (P < 0.01) • Nuts/seeds decreased risk of AML (Women: P < 0.01; Men: P < 0.53) • High intake of dark green vegetables (OR = 0.28, 95% CI: 0.12–0.68), orange vegetables (OR = 0.40, 95% CI: 0.17–0.96), and nuts/seeds (OR = 0.26, 95%CI: 0.11–0.60) decreased the risk of AML in women • Greater consumption of total fruit (OR = 0.26, 95% CI: 0.10–0.69), poultry (OR = 0.28, 95% CI:0.10–0.78), and seafood (OR = 0.39, 95% CI: 0.16–0.96) decreased the risk of AML in men	• People diagnosed with AML were more likely to be obese and had a higher daily caloric intake compared to healthy participants. • People diagnosed with AML consumed higher amounts of white potatoes, more red meat/pork, fewer total vegetables, orange vegetables and dark-green vegetables. • People diagnosed with AML consumed less seafood than healthy participants. • Women diagnosed with AML consumed lower quantities nuts/seeds than healthy participants. • A greater intake of dark green and orange vegetables as well as nuts/seeds may lead to a lower risk of AML for women. • A greater intake of poultry, fruit, and seafood may lead to a lower risk of AML for men.

## Discussion

This scoping review aimed to identify and summarise studies investigating the association between nutrition and the risk for developing AML. Our search identified six primary studies whereas no secondary studies met the inclusion criteria.

One study revealed an association between high intake of red meat and an increased risk for AML [24••]. These observations align with the recommendations of the WCRF’s Diet and Cancer Report, advocating for a diet low in red and processed meats, to mitigate cancer risk. Evidence regarding consumption of red meat and incidence of different cancers is inconsistent, however the most recent large scale meta-analysis identified consumption of red meat to be significantly associated with increased risk of breast cancer, endometrial cancer, colorectal cancer, etc., but reported no results for AML [27]. Furthermore, one of the studies identified that diets characterised by lower consumption of nuts, seeds, vegetables, and seafood were associated with a higher risk of developing AML [25••]. This finding complements the existing body of evidence supporting the protective effects of vegetables, nuts and seeds against overall cancer risk [26, 28–30].

From this review, four studies suggested weak associations between dietary proteins, fish and vegetable consumption and an increased incidence of AML, but these associations were not statistically significant [20••, 21••, 22••, 23••]. Apart from the limited number of articles on this subject, the study type may have contributed to the lack of consistent evidence. The four papers reporting statistically insignificant associations between nutrition and AML incidence utilised large sample sizes [20••, 21••, 22••, 23••]. However, the implementation of cohort and cross-sectional studies, instead of randomised control trials, could have increased the risk of confounders and diminished the ability to establish a causal relationship.

One constituent of many of the food groups mentioned previously, are polyphenols. Polyphenols are a diverse collection of phytochemicals that have been shown to directly inhibit the growth of cancer cells, induce apoptosis and aid in anti-angiogenesis [31]. One such example is the polyphenol quercetin, which has been shown to hinder AML development, via inhibition of CDK2/CDK4 and induction of the tumour suppressor p21 [32]. Many sources of a healthy diet, including fruits (such as berries and grapes), vegetables, legumes, tea, coffee, and red wine are rich in polyphenols. The presence of polyphenols as well as other constituents in the aforementioned food groups may provide an explanation for the protective effects of these groups in reducing the risk for AML.

The study by Li et al. indicated that women with diets high in milk, coffee, beer, and wine were found to have a significantly elevated risk of developing AML. Such associations were found to be statistically insignificant for men [24••]. Likewise, there was a difference in the amount of red meat and vegetables consumed between men and women in the study by Yamamura et al. [25••]. These sex-specific disparities raise questions on whether ‘female sex’ could be a non modifiable risk factor for AML. Hormonal, metabolic and behavioural factors may contribute to differential susceptibility to AML based on diet. Oestrogen has long been implicated in various aspects of haematopoiesis, and oestrogen receptor (ER) signalling has recently been established as a chemotherapeutic target for AML [33]. ER’s interactions with dietary factors could therefore influence AML development differently in women compared to men. Metabolic differences between men and women may also underpin the observed gender-specific associations. For example, women exhibit slower metabolism of alcohol and its byproducts, present in beer and wine, which could potentiate the risk profile it has on AML [34]. The same study identified tea as a protective factor for AML incidence [34]. This finding is consistent with previous research that has identified the polyphenol in green tea, catechin, to possess anti-cancer properties. Such compounds exhibit antioxidant and anti-proliferative properties, potentially mitigating the risk of AML development [35, 36]. Literature has reported coffee to be both protective against certain cancers (liver, colorectal), due to anti-inflammatory properties of the polyphenol, chlorogenic acid, and a risk factor for others (oesophageal, multiple myeloma) [37, 38]. Despite Li et al. [24••] finding coffee to be a risk factor for AML in females, further studies are needed to clarify this association and the specific mechanisms underlying this potential relationship.

The limited focus on investigating the link between nutrition and AML, can be attributed to a number of factors. Firstly, AML though not as rare as some other types of leukemia, still represents a minority of cancer cases, comprising only 1% of all cancers in the United States [1]. As a result it has comparatively received less attention from researchers which may have led to fewer research initiatives compared to more prevalent types of cancer. Moreover, the complex nature of AML pathogenesis involves intricate interactions between genetic, environmental and lifestyle factors which may have prevented researchers from delving into the potential impact of nutrition on disease development. Overall, the multifaceted nature of AML and the presence of established risk factors may have hindered the prioritisation of studies examining the association between nutrition and AML. Multiprofessional healthcare teams including doctors, nutritionists and nurses play a key role in guiding patients on the importance of nutrition in preventing certain cancers, such as AML.Due to their hectic schedules it may be difficult for doctors to dedicate enough time to explain the importance of nutrition in reducing risk for cancer development; therefore other members of the multi-professional healthcare team could contribute to this important task of providing evidence-based guidelines which emphasise a balanced diet rich in fruits, vegetables, whole grains, and lean proteins while minimising processed foods, sugary drinks, and red meat. Additionally, policies should be implemented to support healthcare teams in their efforts to educate patients on cancer prevention through nutrition. These policies can include integrating nutrition education into medical school curricula, increased interdisciplinary collaboration between doctors and nutritionists, and promoting public awareness campaigns. By combining the expertise of healthcare teams and the implementation of supportive policies, patients can be empowered to make informed dietary choices that may reduce their risk of developing cancers like AML.

## Conclusion

This scoping review has presented evidence from two studies reporting an association between diet and the risk of AML, highlighting red meat as a risk factor, and seeds, nuts, vegetables and seafood as potentially protective against the development AML.

Conducting randomised trials to investigate the role of nutrition in AML is challenging due to the insufficient evidence regarding the role of specific dietary factors on the development of AML. Furthermore, AML has a delayed manifestation and ethical concerns arise from exposing individuals to specific diets without a clear basis for potential preventive effects. Considering the limited number of studies exploring the relationship between AML risk and nutrition, future research should prioritise cohort studies, specifically investigating the impact of distinct types of diets and food groups. Cohort studies are often more suitable than randomised trials since they observe participants over time based on their chosen diets and therefore provide valuable insights into associations between nutrition and AML without the ethical challenges associated with an intervention. Therefore, such studies may contribute to a more comprehensive understanding of the role of nutrition in AML risk, providing valuable guidance for policymakers in educating patients about healthy dietary choices.

## Data Availability

No datasets were generated or analysed during the current study.
